# Plasma Prothrombin Time and Esophageal Varices in Patients with Cirrhosis of Liver

**DOI:** 10.5005/jp-journals-10018-1158

**Published:** 2016-07-09

**Authors:** Md Nasirul Islam, Mobin Khan, Nooruddin Ahmad, Mamun-Al-Mahtab, Md Fazal Karim

**Affiliations:** 1 Department of Hepatology, Sir Salimullah Medical College and Mitford Hospital, Dhaka, Bangladesh; 2 The Liver Center, Dhaka, Bangladesh; 3 Department of Hepatology, Bangabandhu Sheikh Mujib Medical University, Dhaka, Bangladesh

**Keywords:** Chronic liver disease, Esophageal varices, Portal hypertension.

## Abstract

**Introduction:**

Cirrhosis of the liver is a common complication of chronic liver disease and is associated with portal hypertension and esophageal varices. In this study, we checked the implication of prothrombin time, if any, in the genesis of esophageal varices.

**Materials and methods:**

Sixty patients with cirrhosis of the liver were randomly assigned into two groups: Group I – 30 cirrhotic patients with esophageal varices, and group II – 30 cirrhotic patients without esophageal varices. The prothrombin time was checked for both groups.

**Results:**

A positive correlation was found between the prolonged plasma prothrombin time (> 4 seconds) and esophageal varices with a sensitivity of 56.67% and specificity of 73.33%. The Child–Pugh score showed a correlation; however, the size of varices did not exhibit any such relation.

**Conclusion:**

Prothrombin time may be cautiously used to assess portal hypertension in a field level and rural setting where endoscopy is not available or feasible.

**How to cite this article:**

Islam MN, Khan M, Ahmad N, Al-Mahtab M, Karim MF. Plasma Prothrombin Time and Esophageal Varices in Patients with Cirrhosis of Liver. Euroasian J Hepato-Gastroenterol 2016;6(1):10-12.

## INTRODUCTION

Cirrhosis of the liver is the most common fatal outcome of chronic hepatitis^1^ and comprises 2.6% of hospital admission.^2^ There are several complications of cirrhosis of the liver; among them the most important and serious is portal hypertension. Manifestations of portal hypertension are esophageal varices, gastric varices, and portal hypertensive gastropathy.^3^ Esophageal varices are formed when portal pressure rises more than 12 mm Hg. The esophageal varices are graded into small, medium, and large. These are responsible for 2 to 9% of upper gastrointestinal bleeding.^4^ Although esophageal varices can be well diagnosed by upper gastrointestinal endoscopy, this is not always possible or feasible in many settings due to cost, availability, and the invasive nature of endoscopic screening.^5^ Different noninvasive modes have been developed to detect esophageal varices;^6,7^ however, more studies may be accomplished to gain more insights into this. Here, we have checked the utility of prothrombin time in predicting esophageal varices in a Bangladeshi setup as a noninvasive approach to accomplish this.

## MATERIALS AND METHODS

A prospective study was done at the Department of Hepatology, Bangabandhu Sheikh Mujib Medical University, within a period extending from January 2010 to December 2011. Sixty patients with liver cirrhosis were enrolled in the study and divided into two groups: Group I comprised 30 cirrhotic patients with esophageal varices and group II comprised 30 cirrhotic patients without esophageal varices. Patients having coagulation disorder/s, those on warfarin therapy, pregnant females, those with contraindication of endoscopy, and those with a history of endoscopic treatment for esophageal varices were excluded from the study. Phytomenadione (vitamin K) 10 mg IV was given for 1 day before the measurement of prothrombin time. Upper gastrointestinal endoscopy was done in all patients. During endoscopy, all patients were assessed for the presence and grades of esophageal varices. The plasma prothrombin time was measured by a coagulation analyzer using human thromboplastin containing calcium (thrombel-s). The severity of the liver disease was assessed by Child–Pugh grading.

Values were expressed either as mean ± SD or in frequency or percentage unless mentioned otherwise. Data were analyzed by using computer-based program Statistical Package for the Social Sciences (SPSS) for Windows version 10. A p-value below 0.05 was considered significant. The correlation between the plasma prothrombin time and esophageal varices was found by the Pearson correlation test.

## RESULT

Out of total 60 patients, 49 were male and 11 were female. Their age ranged from 12 to 79 years and the mean age was 37.11 ± 14.81 years. The demographic, clinical, and laboratory parameters of the patients are shown in Tables 1 and 2. The most common cause of cirrhosis was hepatitis B virus, in 43 cases (71.7%). Other etiologies were hepatitis C virus in 4 cases (6.6%), non-B non-C in 12 cases (20%), and Wilson’s disease in 1 (1.6%) patient. Ascites was found in 29 (96.67%) and 16 (53.33%) of groups I and II patients respectively. Splenomegaly was present in 70 and 56.7% in groups I and II patients respectively.

**Table 1:** **Table 1:** Demographic data of the study group (n = 60)

*Parameter*				*Number of patients* * in group I*		*Number of patients* * in group II*		*Total*		*Percentage*		*p-value*	
Sex		Male		24		25		49		81		NS	
		Female		6		5		11		19		NS	
Age (years)		Range		18–79		12–70		—		—			
		Mean ± SD		37.5 ± 14.7		36.73 ± 15.09		—		—		0.8	
Occupation		Student		1		8		9		15		—	
		Agriculturist		11		6		17		28.4		—	
		Business people		3		5		8		13.3		—	
		Job holder		8		7		15		25		—	
		Manual worker		1		2		3		5		—	
		Housewife		6		2		8		13.3		—	
Etiology		Hepatitis B		20		23		44		71.7		NS	
		Hepatitis C		2		2		4		6.6		NS	
		Non-B non-C		7		5		12		20		NS	
		Wilson’s disease		1		0		1		1.6		—	
Ascites		Present		29 (96.67%)		16 (53.33%)		—		—		< 0.05	
		Absent		1 (3.33%)		14 (46.67%)		—		—		< 0.05	
Splenomegaly		Present		21 (70%)		17 (56.7%)		—		—		< 0.05	
		Absent		9 (30%)		13 (43.3%)		—		—		< 0.05	
Child–Pugh class		A		1 (3.33%)		11 (36.66%)		12		20		< 0.05	
		B		16 (53.33%)		11 (36.66%)		27		45		< 0.05	
		C		13 (43.33%)		8 (26.66%)		21		35		< 0.05	

NS: Not significant

**Table 2:** **Table 2:** Laboratory data of the study group (n = 60)

*Parameter*				*Number of patients* * in group I*		*Number of patients* * in group II*		*Total*		*Percentage*		*p-value*	
ALT		Range		22–328		17–316				—		—	
		Mean ± SD		73.07 ± 66.1		98.8 ± 69.25				—		0.14	
Serum albumin		Range		16–48		20–50				—		—	
		Mean ± SD		28.65 ± 6.85		32.26 ± 8.39				—		0.07	
Prothrombin time		Mean ± SD		18.59 ± 5.19		15.25 ± 2.92				—		0.003	
		< 4 seconds		9 (30%)		22 (73.33%)				—		0.028	
		> 4 seconds		21 (70%)		8 (26.67%)				—		0.011	
Esophageal varices		Small		8		—				26.67		—	
		Medium		12		—				40.0		—	
		Large		10		—				33.33		—	
Gastric varices		Present		1		—				3.33		—	
		Absent		29		—				96.67		—	
Portal hypertensive gastropathy		Absent		11 (36.7%)		27 (90%)				—		—	
		Mild		10 (33.3%)		2 (6.7%)				—		—	
		Severe		9 (30%)		1 (3.3%)				—		—	

Esophageal varices were present in 30 patients: Small sized in 8 (26.67%) patients, medium sized in 12 (40%), and large sized in 10 (33.33%). Red signs over esophageal varices were present in 7 patients (23.33%). Gastric varices were present in 1 (3.33%) patient. Portal hypertensive gastropathy was present in 19 (63%) patients: Mild grade in 10 (33.33%) patients and severe grade in 9 (30%). Both esophageal varices and portal hypertensive gastropathy were present in 19 (63%) patients.

The control value of plasma prothrombin time was 12 seconds. A prolongation of 4 seconds or more was considered as abnormal prothrombin time. In study group I, 17 (56.67%) patients had prolonged prothrombin time (elongation of more than 4 seconds). On the contrary, in study group II, 8 (26.67%) patients had prolonged prothrombin time. Medium-sized esophageal varices were more common in patients with prolonged prothrombin time. Medium-sized esophageal varices were present in 12 patients and the mean prothrombin time was 18.37 seconds. Large-sized esophageal varices were present in 10 patients and the mean prothrombin time mean was 18.82 seconds. R value was 0.195 and p-value was 0.145. So the size of the varices did not correlate with the prothrombin time.

## DISCUSSION

Cirrhosis of the liver is manifested by the development of esophageal varices, gastric varices, portal hypertensive gastropathy, ascites, and splenomegaly. There are several diagnostic tools for the detection of portal hypertension. Direct portal pressure measurement is an invasive procedure. So a minimum invasive procedure like endoscopy of the upper gastrointestinal tract is preferred. It detects the development of portal hypertension by observing the esophageal varices, gastric varices, and portal hypertensive gastropathy in the upper gastrointestinal tract. The grade of esophageal varices often correlates with the severity of the liver disease. Studies have reported that up to 85% of individuals with Child–Pugh C cirrhosis have varices as compared to only 45% those with Child–Pugh A cirrhosis.^8^ On the contrary, plasma prothrombin time is one of the coagulation tests that detects the coagulation defect and certain diseases like chronic parenchymal liver diseases. Study has also shown that prothrombin time could be a predicator for large esophageal varices (LEV) in selected patients with a spleen width of ≤ 44.5 mm and a portal vein diameter of > 11.75 mm.^9^ Although upper gastrointestinal endoscopy remains the gold standard for the diagnosis of gastroesophageal varices, the present study reveals that the measurement of prothrombin time may be of some importance for the assessment of portal hypertension in some patients with cirrhosis of the liver.
